# Two-port laparoscopic anterior resection through a self-made glove device versus conventional laparoscopic anterior resection for rectal cancer: a comparison of short-term surgical results

**DOI:** 10.1186/s12957-016-1029-8

**Published:** 2016-10-26

**Authors:** Hong Zhang, Yunzhi Ling, Jinchun Cong, Mingming Cui, Dingsheng Liu, Chunsheng Chen

**Affiliations:** Department of Colorectal Surgery, Shengjing Hospital, China Medical University, No. 36 SanHao St, Heping District, Shenyang, Liaoning 110004 China

**Keywords:** Reduced-port laparoscopic surgery, Glove port, Anterior resection, Rectal cancer

## Abstract

**Background:**

The laparoscopic approach has become increasingly incorporated into the development of new surgical procedures. An ever-increasing number of surgeons desire methods that minimize surgical trauma and provide improved cosmetic outcomes. Since 2014, we have performed two-port laparoscopic surgery using a transumbilical multichannel glove port and a 12-mm port. The aim of this study was to compare the short-term surgical results of two-port laparoscopic anterior resection (TPLAR) with those of conventional laparoscopic anterior resection (CLAR) for rectal cancer.

**Methods:**

Between January 2014 and May 2014, a total of 27 patients underwent TPLAR and 30 patients underwent CLAR for the treatment of rectal cancer. The short-term surgical results of these two groups of patients were analyzed retrospectively.

**Results:**

The differences in operative time, blood loss, conversion rate, complication rate, distal resection margin, number of harvested lymph nodes, duration until ambulation, duration until first flatus, length of postoperative hospital stay, and overall hospital costs between the two groups were not significant. The median (range) length of the abdominal incisions of the TPLAR patients was shorter than the length of the CLAR patients (5.1 (4.5–16.3) cm vs 8.2 (7.0–10.0) cm, respectively; *p* < 0.001). The respective median (range) postoperative pain scores were lower in the TPLAR than in the CLAR patients at 24 h (4 (1–6) h vs 5 (2–8) h; *p* = 0.045), 48 h (3 (1–4) h vs 4 (range 1–8) h; *P* = 0.004) and 72 h (1 (0–3) h vs 2 (1–5) h; *p* = 0.010). The median overall score on the satisfaction-with-abdominal-incision questionnaire of the TPLAR patients was significantly higher (better) than the score of the CLAR patients.

**Conclusions:**

TPLAR for rectal cancer is safe and feasible, with short-term perioperative and oncological outcomes similar to those of CLAR. TPLAR provides less postoperative pain and better cosmetic outcomes.

## Background

Laparoscopic surgery is widely used for surgical procedures. Procedures using the laparoscopic approach have even become the standard for many abdominal operations, because of the benefits of reduced blood loss, faster postoperative recovery, shorter hospital stay, reduced postoperative pain, and improved cosmetic outcomes compared with open surgery [1–5].

Colorectal cancer is a common malignant tumor, and surgical resection remains the primary method of treatment. Ongoing improvements in laparoscopic techniques have led to the increasing use of laparoscopic surgery for the treatment of colorectal cancer. Many studies have confirmed that laparoscopic surgery provides oncological outcomes comparable to those of open surgery [6–8].

Conventional laparoscopic colorectal surgery is usually performed with five ports. In the pursuit of minimal invasiveness, colorectal surgeons modified the five-port procedure by developing reduced-port laparoscopic surgery (RPLS) and single-incision laparoscopic surgery (SILS). However, SILS can be challenging to perform, requiring specially designed instruments and having a long learning curve. RPLS is a compromise and appears to be suitable for a variety of applications. In addition, SILS and RPLS usually employ a transumbilical incision for the placement of a specific multichannel device for the insertion of instruments and the laparoscope. Most commercially available multichannel devices are expensive. Here, we introduce a simple, inexpensive, and easily available self-made device that consists of a wound retractor and a surgical glove.

The aim of this study was to compare the short-term surgical results of two-port laparoscopic anterior resection (TPLAR) performed via a transumbilical multichannel glove port and a 12-mm port vs conventional laparoscopic anterior resection (CLAR) for rectal cancer.

## Methods

### Study design and patients

This was a retrospective study of patients with rectal cancer who were treated at the Department of Colorectal Surgery of the Shengjing Hospital of China Medical University. Between January 2014 and May 2014, a total of 73 patients underwent surgery for rectal cancer. Patients who had nonmetastatic rectal cancer and required anterior resection were included. The exclusion criteria included the following: (1) preoperative magnetic resonance imaging (MRI) showing a tumor of >4 cm in diameter, evidence of local invasion, or T_4_ cancer; (2) American Society of Anesthesiologists (ASA) classes IV and V; (3) anticipated need of intensive care; (4) history of previous major abdominal surgery; (5) computed tomography (CT) or MRI evidence of tumor infiltration of adjacent organs. A total of 57 patients fulfilled the criteria. Of them, 27 patients underwent TPLAR via a 12-mm port and a transumbilical multichannel port constructed from a wound retractor and a surgical glove. They were matched by age, gender, body mass index (BMI), ASA class, history of abdominal surgery, tumor location, and preoperative TNM stage with 30 patients who underwent CLAR.

All patients underwent a preoperative diagnostic workup that included digital rectal examination, complete colonoscopy, serum carcinoembryonic antigen level, and tumor staging (by contrast-enhanced abdominal CT and pelvic MRI with intravenous contrast) [9]. During colonoscopy, tumor samples were obtained from each patient for histopathological examination. None of the patients received neoadjuvant chemoradiation therapy. Written informed consent was obtained from each patient prior to surgery, and the study was approved by the ethics committee of Shengjing Hospital of China Medical University.

### Surgical technique

**Fig. 1:**
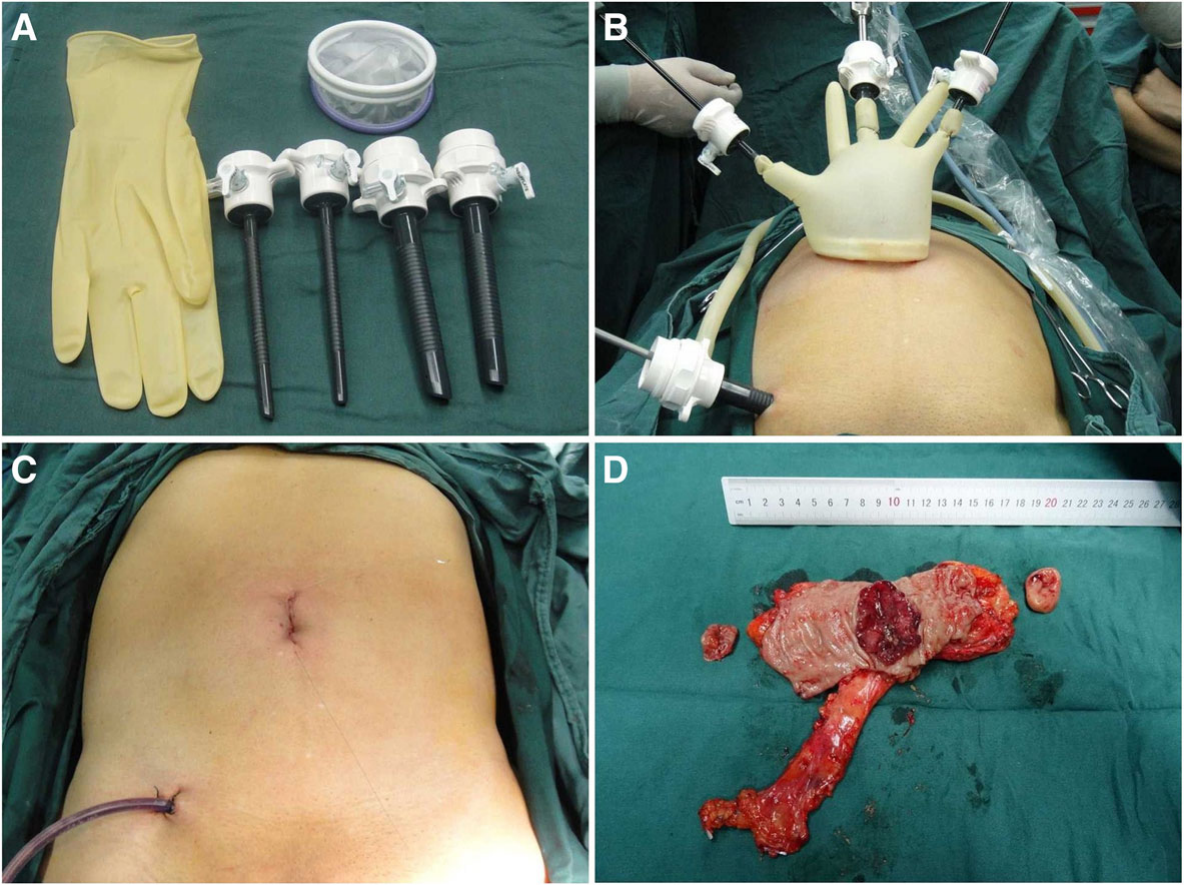
Two-port laparoscopic anterior resection (TPLAR) using a 12-mm port and a glove port for rectal cancer: a surgical glove, wound retractor, and trocars were required for the self-made device (**a**). The self-made device was inserted into the transumbilical incision. An additional 12-mm trocar was placed into an incision at McBurney’s point (**b**). A drain was placed through the additional port incision at McBurney’s point. The transumbilical incision was closed (**c**). Representative image of specimen from a patient undergoing TPLAR that was extracted via the transumbilical incision (**d**)

All the procedures were performed by the same experienced surgical team. Under general anesthesia, all patients were placed in the modified lithotomy position. For TPLAR, a 3–5-cm transumbilical incision was made, and a self-made transumbilical multichannel device that was constructed from a wound retractor (Yunlong Medical Instrument; Wuxi Yunlong Medical Instrument Co., Ltd., Wuxi, China) and a surgical glove (Fig. 1a) was inserted into the transumbilical incision. Two 5-mm trocars (Tyco Healthcare Group LP, Norwalk, CT, USA) and a 12-mm trocar (Versaport™ PlusV2 bladed trocars; Covidien PLC, Mansfield, MA, USA) were then inserted into the little finger, thumb, and middle finger, respectively, of the glove. An additional 12-mm trocar was placed in an incision at McBurney’s point. A rigid 30°, 10-mm laparoscope was inserted into the 12-mm trocar at the transumbilical port (Fig. 1b). The operation was performed using conventional laparoscopic instruments. An ultrasonic scalpel (Ethicon Endo-Surgery, LLC; Guaynabo, Puerto Rico, USA) was used for dissection, and the medial-to-lateral approach was used for dissecting the right sigmoid mesentery. The inferior mesenteric vessels were skeletonized, and high ligation was performed by Hem-o-lok clips (Teleflex Medical; Research Triangle Park, NC, USA) at the root of each vessel. All patients underwent a procedure based on the principle of total mesorectal excision. An endoscopic linear stapler (Endo GIA™ Ultra Universal Stapler with Purple Medium/Thick Cartridges; Covidien PLC, Mansfield, MA, USA) was inserted into the pelvic cavity through the additional port, and the rectum was then transected approximately 3 cm from the lower margin of the tumor by the endoscopic linear stapler. The specimen was extracted via the transumbilical incision. After insertion of the anvil, the anastomosis was performed by an EEA circular stapler (Premium Plus CEEA™; Covidien PLC, Mansfield, MA, USA). After the procedure, a drain was placed through the incision at McBurney’s point, and the transumbilical incision was closed (Fig. 1c). The lengths of the transumbilical incision and the incision at McBurney’s point were measured. Fig. 1d shows a photograph of a representative specimen.

For CLAR, the procedures were performed via five ports, and most procedures were similar to those used for TPLAR; however, the specimen was extracted through an enlarged umbilical incision.

### Postoperative care

All patients were treated by the same standardized protocol after surgery. Each patient was allowed to drink 50 mL of water per day from the first day after surgery until passage of flatus and then was allowed unlimited oral intake of fluids. The patient was allowed a solid diet after the passage of feces. Each patient chewed gum three times daily from the morning of postoperative day 1 until the first passage of stool. Patients were given postoperative intravenous antibiotics for 3–5 days. We encouraged early ambulation and moderate activity. Postoperative analgesia consisted of intravenous parecoxib sodium 40 mg every 12 h for 3 days.

### Satisfaction-with-abdominal-incision questionnaire

Satisfaction-with-abdominal-incision questionnaire (SAIQ) is a five-item questionnaire that is used to assess the patient’s satisfaction with the abdominal incisions (Table 1). These items included position, length, esthetics, healing, and pain at the incision site. A possible score for each item ranged from “1” for “not at all” to “4” for “very much.” The scores were summed for a total overall score ranging from 5–20. A higher score represented a higher degree of satisfaction with the abdominal incisions. The scores of each individual item were compared between the TPLAR and CLAR patients.

**Table 1:** Satisfaction-with-abdominal-incision questionnaire

	Not at all	A little	Quite a bit	Very much
1. Are you satisfied with the position of the abdominal incisions	1	2	3	4
2. Are you satisfied with the length of the abdominal incisions	1	2	3	4
3. Are you satisfied with the esthetics of the abdominal incisions	1	2	3	4
4. Are you satisfied with the healing of the abdominal incisions	1	2	3	4
5. Are you satisfied with the pain associated with the abdominal incisions	1	2	3	4

### Data collection

The following types of data were analyzed: patient and tumor characteristics, procedural details and perioperative data, postoperative pain score, length of postoperative hospital stay, and SAIQ scores. Postoperative pain was measured using a visual analogue scale (VAS) at 12, 24, 48, 72, 96, and 120 h after surgery. Postoperative complications included bleeding, anastomotic leakage, wound infection, intraabdominal abscess, ileus, and urinary retention. All patients were asked to complete the SAIQ, which was administered by a nurse, 1 day before or the day of discharge from the hospital. The overall direct medical costs were calculated for each patient group. The patients were followed by the outpatient department at 1-month intervals for the first 3 months after discharge from the hospital and at 3-month intervals thereafter for longer than 1 year. We contacted some patients by phone to confirm the validity of the follow-up data.

### Statistical analysis

Results are expressed as median (range) or rates (%). Continuous variables were analyzed using the Mann-Whitney *U* test. Discrete variables were compared by the chi-square test or Fisher exact test. All statistical calculations were performed using SPSS software, version 19.0 (SPSS Inc., Chicago, IL, USA). A *p* value ≤0.05 was considered statistically significant.

## Results

### Patient and tumor characteristics

Fifty-seven patients with primary rectal cancer, who underwent anterior resection between January 2014 and May 2014, were enrolled in our study (27 TPLAR and 30 CLAR patients). Patient and tumor characteristics are presented in Table 2. The differences in age, gender, BMI, ASA class, history of previous abdominal operation, tumor distance from anal verge, and preoperative TNM stage between the two patient groups were not statistically significant (*p* > 0.05 for all comparisons).

**Table 2:** Patient and tumor characteristics

	TPLAR (*n* = 27)	CLAR (*n* = 30)	*p* value
Age (years), median (range)	60 (43–71)	61 (49–72)	0.517
Gender			0.462
Male, *n* (%)	17 (63.0)	16 (53.3)	
Female, *n* (%)	10 (37.0)	14 (46.7)	
BMI (kg/m^2^), median (range)	24.2 (18.6–33.2)	24.7 (17.2–29.7)	0.911
ASA class			0.930
ASA 1, *n* (%)	6 (22.2)	7 (23.3)	
ASA 2, *n* (%)	16 (59.3)	19 (63.3)	
ASA 3, *n* (%)	5 (18.5)	4 (13.4)	
Previous abdominal operation *n* (%)	3 (11.1)	5 (16.7)	0.709
Tumor distance from anal verge (cm), median (range)	8 (5–12)	7 (5–12)	0.538
Preoperative TNM stage			0.665
I, *n* (%)	3 (11.1)	2 (6.7)	
II, *n* (%)	11 (40.7)	10 (33.3)	
III, *n* (%)	13 (48.2)	18 (60.0)	

*TPLAR* two-port laparoscopic anterior resection, *CLAR* conventional laparoscopic anterior resection, *BMI* body mass index, *ASA clas*s American Society of Anesthesiologists class

### Procedural details and perioperative outcomes

Procedural details and perioperative outcomes are provided in Table 3. The respective median (range) operative times were longer in the TPLAR group than the CLAR group (120 (100–160) min vs 115 (80–140) min), but the difference was not statistically significant (*p* = 0.099). The difference in median volumes of blood lost was not statistically significant (*p* = 0.084). One TPLAR patient was converted to open surgery because of abdominal adhesions, and no patients were converted to conventional laparoscopic surgery. No CLAR patients were converted to open surgery. The difference in rates of open surgical conversion was not statistically significant (*p* = 0.474). No intraoperative complications occurred during any of the procedures. All of the abdominal incisions, including transumbilical incision, trocar sites, enlarged umbilical incision, and open surgical incision were measured. The median length of the abdominal incision was statistically significantly shorter in the TPLAR group than in the CLAR group (5.1 (4.5–16.3) cm vs 8.2 (7.0–10.0) cm; *p* < 0.001). The differences in the median width of the distal resection margins (*p* = 0.993), median number of harvested lymph nodes (*p* = 0.086), and the percentages of patients with each TNM stage (*p* = 0.488) were not statistically significant. The overall postoperative complication rate was 3.7 % in the TPLAR group and 10 % in the CLAR group (*p* = 0.613). One TPLAR patient developed postoperative urinary retention, and three CLAR patients developed postoperative complications (ileus (*n* = 1), urinary tract infection (*n* = 1), and urinary retention (*n* = 1)). None of the patients with complications underwent additional surgery for their postoperative complication. The differences in median duration until ambulation (*p* = 0.261), duration until first flatus (*p* = 0.243), length of postoperative hospital stay (*p* = 0.060), and overall hospital costs (*p* = 0.676) between the two groups of patients were not statistically significant. None of the patients in either group was rehospitalized during the first 30 postoperative days. In addition, none of the patients developed hernia at the trocar sites, metastasis to the incisions, or local recurrences. None of the patients died during the follow-up period.

**Table 3:** Procedural details and perioperative data

	TPLAR (*n* = 27)	CLAR (*n* = 30)	*p* value
Duration of procedure (min), median (range)	120 (100–160)	115 (80–140)	0.099
Blood loss (mL), median (range)	30 (10–60)	40 (10–70)	0.084
Conversion to open surgery, *n* (%)	1 (3.7)	0 (0.0)	0.474
Intraoperative complication, *n* (%)	0 (0.0)	0 (0.0)	
Length of abdominal incision (cm), median (range)	5.1 (4.5–16.3)	8.2 (7.0–10.0)	<0.001
Distal resection margin (cm), median (range)	2 (1.7–2.3)	2 (1.6–2.5)	0.993
No. of harvested lymph nodes, median (range)	15 (11–19)	16 (11–23)	0.086
Postoperative TNM stage			0.488
I, *n* (%)	2 (7.4)	1 (3.3)	
II, *n* (%)	11 (40.7)	9 (30.0)	
III, *n* (%)	14 (51.9)	20 (66.7)	
Reoperation, *n* (%)	0 (0.0)	0 (0.0)	
Postoperative complication, *n* (%)	1 (3.7)	3 (10.0)	0.613
Duration until ambulation (h), median (range)	18 (15–22)	17 (10–26)	0.261
Duration until first flatus (h), median (range)	51 (39–63)	52.5 (42–240)	0.243
Length of postoperative hospital stay (d), median (range)	9 (7–13)	8 (7–21)	0.060
Overall hospital costs ($), median (range)	6366 (5892–7465)	6711 (5983–7679)	0.676
Readmission, *n* (%)	0 (0.0)	0 (0.0)	

*TPLAR* two-port laparoscopic anterior resection, *CLAR* conventional laparoscopic anterior resection

### Postoperative pain scores

**Fig. 2:**
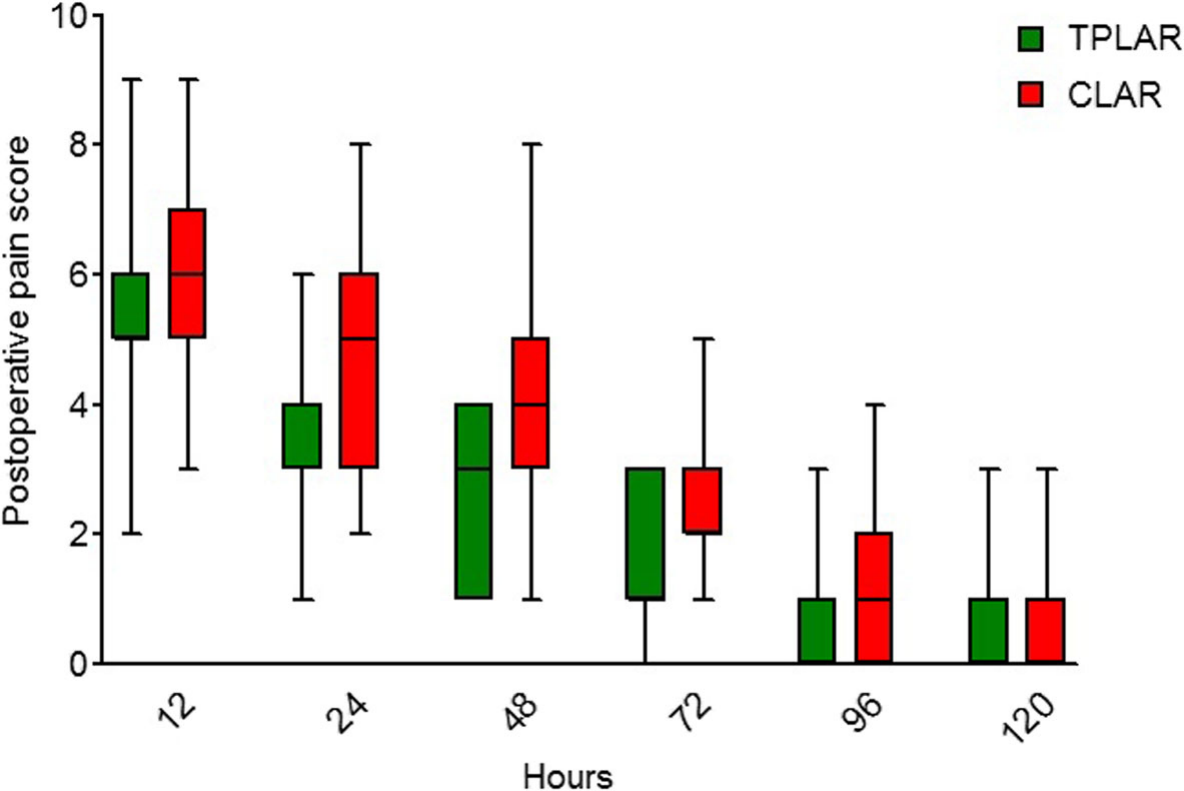
Postoperative pain scores of patients undergoing two-port laparoscopic anterior resection (TPLAR) or conventional laparoscopic anterior resection (CLAR). Postoperative pain scores at 24, 48, and 72 h after surgery were lower (better) in the TPLAR than those in the CLAR patients (*p* < 0.05)

The postoperative pain scores of both TPLAR and CLAR patients dropped rapidly (Fig. 2). Table 4 summarizes the postoperative pain scores of the two patient groups. The respective median (range) postoperative pain scores of the TPLAR patients were statistically significantly lower (less pain) than those of the CLAR patients at 24 h (4 (1–6) h vs 5 (2–8) h; *p* = 0.045), 48 h (3 (1–4) vs 4 (1-8) h; *p* = 0.004), and 72 h (1 (0–3) h vs 2 (1-5); *p* = 0.010). The differences in postoperative pain scores were not statistically significant at 12, 96, and 120 h.

**Table 4:** Postoperative pain score

	TPLAR (*n* = 27)	CLAR (*n* = 30)	*p* value
12 h, median (range)	5 (2–9)	6 (3–9)	0.067
24 h, median (range)	4 (1–6)	5 (2–8)	0.045
48 h, median (range)	3 (1–4)	4 (1–8)	0.004
72 h, median (range)	1 (0–3)	2 (1–5)	0.010
96 h, median (range)	0 (0–3)	1 (0–4)	0.091
120 h, median (range)	0 (0–3)	0 (0–3)	0.182

*TPLAR* two-port laparoscopic anterior resection, *CLAR* conventional laparoscopic anterior resection

### Satisfaction-with-abdominal-incision questionnaire

**Fig. 3:**
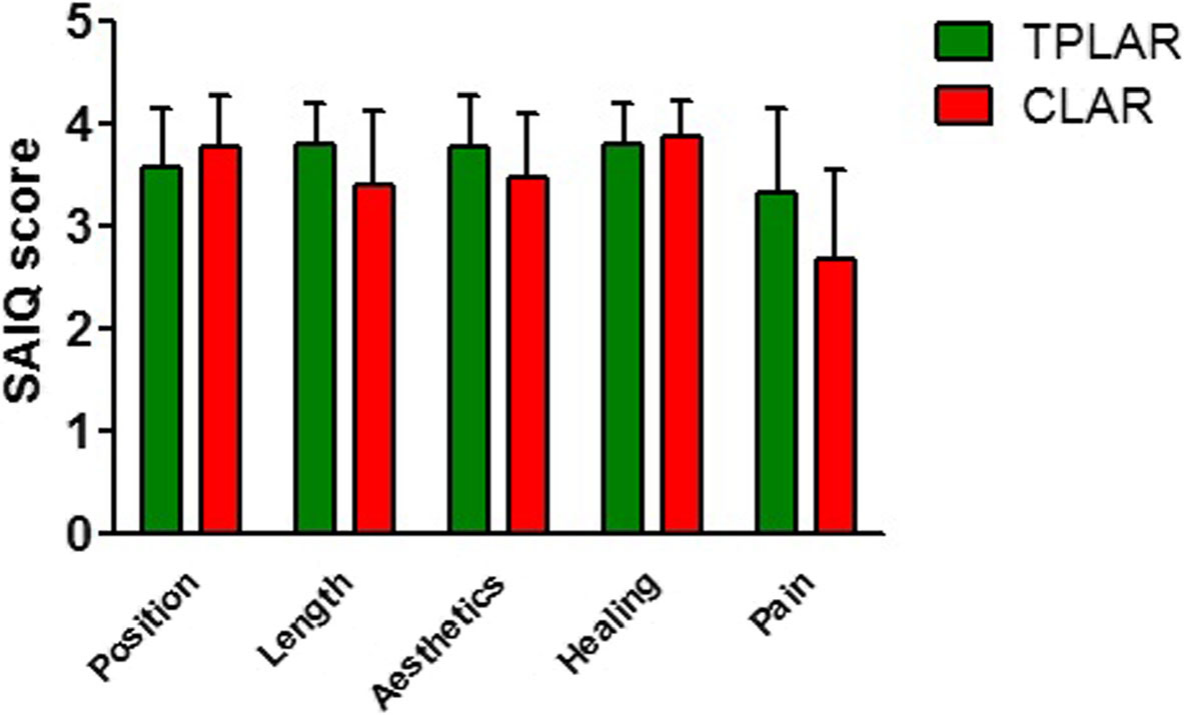
Satisfaction-with-abdominal-incision questionnaire (SAIQ) scores of patients undergoing two-port laparoscopic anterior resection (TPLAR) or conventional laparoscopic anterior resection (CLAR). The scores for length, esthetics, and incisional pain were higher (greater satisfaction) in the TPLAR than those in the CLAR patients (*p* < 0.05)

The outcomes of the SAIQ are detailed in Table 5. The overall satisfaction with abdominal incisions was greater among the TPLAR patients than among the CLAR patients (*p* = 0.001). The differences between patient groups in the median scores for length (*p* = 0.017), esthetics (*p* = 0.030), and pain (*p* = 0.003) were statistically significant. However, the differences in the median scores for position and healing of incisions were not significant (Fig. 3).

**Table 5:** SAIQ outcomes

	TPLAR (*n* = 27)	CLAR (*n* = 30)	*p* value
Position of incisions, median (range)	4 (2–4)	4 (2–4)	0.171
Length of incisions, median (range)	4 (3–4)	4 (2–4)	0.017
Esthetics of incisions, median (range)	4 (2–4)	4 (2–4)	0.030
Healing of incisions, median (range)	4 (3–4)	4 (3–4)	0.595
Pain at incisions, median (range)	4 (1–4)	3 (1–4)	0.003
Overall satisfaction (sum of all scores)	19 (12–20)	17 (13–20)	0.001

*SAIQ* satisfaction-with-abdominal-incision questionnaire, *TPLAR* two-port laparoscopic anterior resection, *CLAR* conventional laparoscopic anterior resection

## Discussion

Laparoscopic surgery for rectal cancer has been performed worldwide. The COLOR II trial reported that laparoscopic surgery provided rates of locoregional recurrence and disease-free and overall survival similar to those of open surgery [6]. The procedures used in conventional laparoscopic rectal cancer surgery are usually performed through five ports. Such multiport laparoscopic surgeries may lead to worse cosmetic outcomes and increased rates of trocar-related complications, including hernias, wound infection, and cancer recurrence at the sites of the ports. Therefore, some less invasive laparoscopic surgical techniques have been introduced. SILS has been reported to lead to greatly decreased wound pain, improved cosmesis, decreased length of hospital stay, and reduced rate of postoperative complications [10–12]. However, because of the shortcomings of SILS, including cross-hand phenomenon and limited triangulation of the scope and tissue, performing dissection and exposure of colorectal tissue is difficult. The operating surgeon usually has a prolonged learning curve for this technique [13, 14]. In addition, the study by Lim et al. found that the operative time was significantly longer for SILS than for multiport laparoscopic surgery [15]. The widespread adoption of SILS for colorectal cancer is not probable. Therefore, a laparoscopic procedure that combines a single incision with an additional port might be an acceptable compromise that leads to a decreased learning curve while still providing improved cosmetic outcomes over conventional laparoscopic surgery.

Several authors have reported that RPLS for colorectal cancer is feasible and safe [16–20]. In general, their studies found that operative results for RPLS and CLAR were similar, including blood loss, conversion rate, number of harvested lymph nodes, size of the distal resection margin, complication rates, and duration of hospital stay. The surgical procedures performed via RPLS may be more difficult to perform than with conventional laparoscopic surgery, but only one study reported RPLS was significantly more time consuming [17].

In some of the previous studies, most of the surgeons made a transumbilical incision for the placement of a commercially available multichannel system, and then an additional 5- or 12-mm trocar was placed in an incision in the right lower quadrant [16–18]. However, these commercially available devices may lead to increased costs. We used a two-port technique that consisted of a transumbilical multichannel port, which was created from a wound retractor and a surgical glove, plus an additional port for a 12-mm trocar. Our self-made multichannel device might lead to somewhat reduced costs. In addition, our device also provided some other advantages. We inserted trocars into the fingers of the glove, which allowed a wider range of movement for our instruments than the movement allowed by commercially available devices. Our self-made multichannel device also allows the addition of one to two instruments at any time, if needed; and the wound retractor incorporated into the device can be rolled optionally to accommodate different sizes of patients and can help prevent infections and metastasis at the site of the transumbilical incision associated with extraction of the specimen. A potential disadvantage of this self-made device might be leakage of gas, but it rarely happens and can be easily resolved during surgery. We are not the first surgeons to use this laparoscopic glove-port technique. Reports published in the field of minimally invasive surgery include those on glove-port splenectomy, appendectomy, adrenalectomy, cholecystectomy, hysterectomy, and gastric wedge resection [21–26].

A total of 27 patients underwent TPLAR for rectal cancer in our hospital at the time of this report. We evaluated and compared the short-term surgical results of the patients undergoing TPLAR with the results of 30 patients who underwent CLAR for rectal cancer. The operative outcomes, including operative time, blood loss, conversion rate, complication rate, and postoperative recovery were similar between the two patient groups. The oncological adequacy of TPLAR was a concern. However, in our study, the differences in sizes of distal resection margin and number of harvested lymph nodes were not significant. In addition, none of the patients in either group developed local recurrence or died during the follow-up period. Our results demonstrate that TPLAR for rectal cancer can provide satisfactory short-tem oncological outcomes, which were comparable to those reported by Bae et al. [16].

Our study found that the mean length of abdominal incisions in the TPLAR group was significantly shorter than the mean length in the CLAR group. Lim et al. reported that differences between the lengths of umbilical incisions resulting from umbilical-incision laparoscopic colorectal cancer surgery using a single additional port and from conventional laparoscopic surgery were not significant [17]. The self-made multichannel device can be placed into a 3–3.5-cm incision, and the resected specimen is usually extracted through the umbilical incision, which partly determines the length of the umbilical incision. However, CLAR procedures, which are performed through five ports, result in an overall increase in the total length of the incisions.

Three case-matched studies [10, 27, 28] and two randomized controlled trials [29, 30] have compared SILS and conventional laparoscopic surgery for colorectal cancer. In general, pain after SILS was less than pain after CLAR. Lim et al. [17] and Kawamata et al. [18] reported case-matched studies that compared two-port laparoscopic surgery and conventional laparoscopic surgery for colorectal cancer but did not report the outcomes on postoperative pain. Our study used a VAS to assess postoperative pain at different time points after surgery. The TPLAR patients had less postoperative pain at 24, 48, and 72 h than the CLAR patients. These results suggest that the number of incisions probably affects the degree of postoperative pain, and a lower number of incisions might result in less postoperative pain. Interestingly, the duration of postoperative recovery was similar between the two groups of patients, despite the decreased postoperative pain of the TPLAR patients.

To the best of our knowledge, there have not been any reports on the use of a gold-standard scoring system for evaluating the degree of patient satisfaction with abdominal incisions until now. Arrigoni et al. reported on a quality-of-life questionnaire using a five-point Likert scale that included the determination of patient satisfaction with wound cosmesis [31]. However, this scoring system was only a rough evaluation of cosmetic outcomes. We created a detailed, five-item questionnaire that assessed patient satisfaction with the position, length, esthetics, healing, and pain of the incisions. Our results showed that patients undergoing either TPLAR or CLAR for rectal cancer assigned high scores for each item, suggesting that both procedures provided abdominal incisions that were highly satisfactory to patients. Moreover, the extent of overall satisfaction and satisfaction with the length, esthetics, and incisional pain was higher in the TPLAR than that in the CLAR patients. The visible length of the umbilical incisions was reduced over the time of wound healing, and the transumbilical incisions were often invisible by week 1 after surgery. Therefore, the scars of the lateral ports became the major factor associated with the degree of patient satisfaction. The multiple postoperative scars resulting from CLAR might account for the decreased satisfaction of CLAR patients.

The placement of a tube into the pelvic cavity after rectal surgery in order to drain potential postoperative blood loss and anastomotic leakage is controversial. Notably, pelvic drainage is not recommended by the Society of Enhanced Recovery After Surgery [32]. However, we routinely place a drainage tube in the pelvic cavity because we think that this practice allows easy monitoring for anastomotic leakage, which is manifested by changes in the appearance of the drainage contents. Although the use of a drainage tube after elective colorectal surgery has not appeared to prevent anastomotic leakage, the tube allows the timely diagnosis and treatment of anastomotic leakage [33]. Moreover, the drainage tube can be used for irrigation and drainage of rectal anastomotic leakage. With SILS, a drainage tube cannot be placed into the iliac fossa, but it can be placed into the pelvic cavity through the additional port incision used in TPLAR. Therefore, a two-port laparoscopic surgery is considered safer than single-port laparoscopy.

This study has limitations. First, the numbers of patients in the two groups were small. Second, our study had a short follow-up time. We therefore could not evaluate the long-term oncological results and determine the incidence of late complications, such as trocar-site hernia and metastasis to the incisional sites for TPLAR vs CLAR. And third, the colorectal surgeons in this study had performed more than 500 CLARs for rectal cancer before they began using two-port procedures. The difference in proficiency between the two techniques might have led to a small bias.

## Conclusions

In conclusion, two-port laparoscopic technology can overcome some of the shortcomings of conventional laparoscopic surgery and SILS, while also providing some of their benefits. TPLAR for rectal cancer, as performed by expert laparoscopic surgeons, is safe and feasible. The short-term perioperative and oncological outcomes were similar to those of CLAR. TPLAR provides less postoperative pain and better cosmetic outcomes. In addition, our self-made multichannel device is simple, cheap, and easily available, and can reduce costs. However, additional well-designed randomized studies with larger numbers of patients are needed for evaluating the long-term oncological results of our procedure.

## Abbreviations

ASA: American Society of Anesthesiologists
BMI: Body mass index
CLAR: Conventional laparoscopic anterior resection
CT: Computed tomography
ICU: Intensive care unit
MRI: Magnetic resonance imaging
RPLS: Reduced-port laparoscopic surgery
SAIQ: Satisfaction-with-abdominal-incision questionnaire
SILS: Single-incision laparoscopic surgery
TPLAR: Two-port laparoscopic anterior resection
VAS: Visual analogue scale
